# Efficacy and safety of switching to iGlarLixi from premixed insulin therapy in patients with type 2 diabetes: A real‐world experience

**DOI:** 10.1111/dme.70275

**Published:** 2026-04-01

**Authors:** Chun‐Jui Huang, Shun‐Jie Yang, Cheng‐Pin Cheng, Chun‐Hsing Lin, Ya‐Chun Lee, Harn‐Shen Chen

**Affiliations:** ^1^ Division of Endocrinology and Metabolism, Department of Medicine Taipei Veterans General Hospital Taipei Taiwan; ^2^ School of Medicine, College of Medicine National Yang Ming Chiao Tung University Taipei Taiwan

**Keywords:** iGlarLixi, premixed insulin, type 2 diabetes mellitus

## Abstract

**Aims:**

Fixed‐ratio combinations such as iGlarLixi are recommended over premixed insulin as first‐line injectable therapy for type 2 diabetes mellitus (T2DM), yet real‐world evidence on transitions from premix to iGlarLixi remains limited. This study aimed to evaluate the efficacy and safety of switching from premixed insulin to iGlarLixi in a real‐world clinical setting.

**Methods:**

This retrospective cohort included adults with T2DM who transitioned from premixed insulin to iGlarLixi between July 2020 and July 2023 at Taipei Veterans General Hospital. Glycaemic parameters were collected at baseline, 3 and 6 months after transition.

**Results:**

Forty patients (mean age 67.6 ± 10.5 years) were included. Among those with baseline HbA_1c_ >58 mmol/mol (7.5%, *n* = 28), HbA_1c_ decreased from 79 ± 18 (9.4% ± 1.7%) to 67 ± 18 mmol/mol (8.2% ± 1.6%) at 6 months (*p* = 0.01) and 23.1% achieved HbA_1c_ ≤53 mmol/mol (7.0%). The largest HbA_1c_ reduction was observed in those previously on 40–50 units/day of premix insulin (from 83 ± 21 [9.8% ± 1.9%] to 65 ± 15 mmol/mol [8.1% ± 1.4%], *p* = 0.037). Total insulin dose and injection frequency significantly decreased (*p* < 0.001), while hypoglycaemia incidence dropped from 60.0% to 12.5% (*p* < 0.001). Among patients with pre‐existing hypoglycaemia, glycaemic control remained stable despite reductions in insulin dose and injection frequency (*p* = 0.001, *p* = 0.032, respectively). Higher HOMA‐IR was significantly associated with suboptimal glycaemic response in multivariate model (*p* = 0.037).

**Conclusions:**

Switching from premixed insulin to iGlarLixi is a safe and effective strategy to simplify therapy and improve glycaemic management. Insulin resistance may influence response.

**What's new?**

**What is already known?**

Fixed‐ratio combination therapies such as iGlarLixi are effective for patients inadequately controlled on oral antidiabetic agents or basal insulin. However, real‐world evidence supporting transitions from premixed insulin to iGlarLixi remains limited.

**What this study has found?**

In real‐world clinical practice, switching from premixed insulin to iGlarLixi was associated with improvements in HbA_1c_, reduced hypoglycaemia and lowered total insulin dose and injection burden.

**What are the implications?**

iGlarLixi may represent a feasible and safe de‐escalation option for patients on complex premixed insulin regimens, enabling treatment simplification without compromising glycaemic control.

## 1 INTRODUCTION

Type 2 diabetes mellitus (T2DM) is characterized by progressive declines in β‐cell function and increasing insulin resistance, often necessitating the escalation to injectable therapy as the disease advances.^1^, ^2^ Premixed insulin formulations, which combine basal and prandial components in fixed proportions, have long been favoured for their convenience and simplicity compared with traditional basal–bolus regimens.^3^ In many Asian countries, including Taiwan, premixed insulin is commonly used as an initial insulin regimen to circumvent the complexities of transitioning from basal insulin to premixed insulin when intensification is required.^4^ However, despite its widespread use, glycaemic outcomes with premixed insulin remain suboptimal for many patients due to challenges with dose titration, increased risk of hypoglycaemia and associated weight gain.^5^, ^6^ These challenges highlight the need for alternative injectable strategies that can optimize glycaemic control while reducing treatment burden.

Fixed‐ratio combination injection therapies have emerged as an alternative to premixed insulin regimens and are now recommended as first‐line injectable options prior to the use of premixed insulin.^7^, ^8^ Among these, iGlarLixi, composed of insulin glargine 100 U/mL and the short‐acting glucagon‐like peptide‐1 receptor agonist (GLP‐1 RA) lixisenatide, provides complementary glucose‐lowering mechanisms. Insulin glargine primarily improves fasting glycaemia, whereas lixisenatide attenuates postprandial glucose excursions through delayed gastric emptying and glucose‐dependent insulin secretion.^9^ Compared with premixed insulin, which typically requires twice‐daily administration, iGlarLixi allows a once‐daily administration and has been shown to reduce glycaemic variability in clinical studies.^6^ Clinical trials have demonstrated that iGlarLixi achieves greater HbA_1c_ reduction without increasing the risk of hypoglycaemia or weight gain compared with basal insulin or GLP‐1 RA alone.^10^, ^11^

Traditionally, insulin therapy in T2DM follows a stepwise intensification approach, beginning with a single daily basal insulin injection and intensifying to basal‐plus or basal–bolus regimens as needed.^7^, ^8^ Consequently, there is limited evidence supporting a step‐down approach, such as de‐intensifying from a premixed insulin regimen to a once‐daily fixed‐ratio combination like iGlarLixi, despite its potential benefits in simplifying treatment and reducing hypoglycaemia risk. This study aimed to evaluate the efficacy and safety of switching from premixed insulin to iGlarLixi in a real‐world clinical setting and to identify patient characteristics associated with glycaemic response, with the goal of informing individualized treatment de‐escalation strategies.

## 2 METHODS

### 2.1 Study design

This retrospective cohort study included adults with T2DM who transitioned from a stable premixed insulin regimen to iGlarLixi between July 2020 and July 2023 at Taipei Veterans General Hospital, Taiwan. A stable dose was defined as no change in oral antidiabetic drug (OAD) therapy for at least 3 months before the transition and premixed insulin dose variations within ±4 units during the same period. All switches were made at the discretion of treating clinicians as part of routine diabetes care. Physicians typically recommended iGlarLixi when they anticipated that the fixed‐ratio combination could resolve a specific clinical concern, such as inadequate glycaemic control, hypoglycaemia, weight gain, injection burden, or intolerance to premixed insulin. The initial iGlarLixi dose was individualized based on the patient's prior total daily premixed insulin dose, with the intermediate‐acting component considered a surrogate for the basal insulin dose when determining the conversion. Clinicians further tailored the starting dose according to current fasting glucose values, overall glycaemic control and hypoglycaemia risk. Subsequent titration generally followed the routine clinical practice approach of adjusting by approximately 2 units every 3 days to achieve a fasting glucose target of 5.6–7.2 mmol/L (100–130 mg/dL), but all dose modifications remained individualized. Patients were typically reassessed about 2 weeks after switching, with follow‐up intervals thereafter determined by clinical judgement. Patients younger than 18 years, those who discontinued iGlarLixi within 3 months, or those lost to follow‐up during that period were excluded. The study was approved by the local Institutional Review Board and conducted in accordance with the Declaration of Helsinki.

### 2.2 Data collection

Demographic, clinical and biochemical data were retrieved from the electronic medical records of Taipei Veterans General Hospital. Glycaemic parameters, including fasting plasma glucose (FPG) and glycated haemoglobin (HbA_1c_), were collected at baseline and approximately 3 and 6 months after the transition. Hypoglycaemia history was defined as any documented episode occurring within the 12‐month period prior to switching from premixed insulin. Events were identified retrospectively from electronic medical records, including recorded glucose values <3.9 mmol/L (70 mg/dL) and clinician‐documented or patient‐reported compatible symptoms. Hypoglycaemic events during the 6‐month follow‐up after switching were similarly identified using the same data sources. In patients with paired measurements of FPG and fasting C‐peptide, endogenous insulin resistance was estimated using a modified homeostasis model assessment (HOMA‐IR), calculated as: 1.5 + FPG (mmol/L) × c‐peptide (pmol/L)/2800.^12^ Additional biochemical indices relevant to diabetes complications, including liver and kidney function tests and lipid profiles, were also collected. Information on insulin dose, injection frequency and concomitant OAD use was recorded.

### 2.3 Statistical analysis

All statistical analyses were performed using IBM SPSS Statistics (version 25.0; IBM Corp., Armonk, NY, USA). Continuous variables were expressed as mean ± standard deviations and categorical variables as counts (percentages). Normality was assessed using the Shapiro–Wilk test. Several variables, including HbA_1c_ at 6 months, injection frequency at all time points, total insulin dose at 3 and 6 months and the iGlarLixi initial dose, did not follow a normal distribution (*p* < 0.05); therefore, non‐parametric tests were applied. The Wilcoxon signed‐rank test was used for within‐group comparisons, whereas the Mann–Whitney *U* or Fisher's exact test was applied for between‐group comparisons. Paired categorical changes (e.g., HbA_1c_ goal attainment, hypoglycaemia incidence and OAD use) were assessed using McNemar's test. Logistic regression analyses were performed to identify predictors of suboptimal glycaemic response at 6 months. Variables with *p* < 0.20 in univariate analyses were included in multivariate model. A two‐tailed *p* value <0.05 was considered statistically significant. Subgroup analyses were exploratory in nature, and no formal interaction terms were tested due to limited sample size.

## 3 RESULTS

A total of 44 patients were identified; four were excluded due to loss to follow‐up or switching to another injectable therapy within 3 months. The remaining 40 patients constituted the analytic cohort. Of these, 70.0% (*n* = 28) transitioned to iGlarLixi due to suboptimal glycaemic control (HbA_1c_ >58 mmol/mol [7.5%]), while 12 patients (30.0%) switched because of hypoglycaemia (*n* = 10, 83.3%) or to reduce injection frequency (*n* = 2, 16.7%).

### 3.1 Baseline characteristics

The characteristics of the study population are summarized in Table 1. The mean age of the patients was 67.6 ± 10.5 years, with an average body mass index (BMI) of 27.3 ± 4.6 kg/m^2^ and a mean diabetes duration of 18.4 ± 10.0 years. Baseline FPG and HbA_1c_ were 8.3 ± 2.8 mmol/L (148.5 ± 50.7 mg/dL) and 69 ± 22 mmol/mol (8.5 ± 2.0%), respectively, on an average premixed insulin dose of 43.7 ± 22.0 units/day. Before transitioning, the premixed insulin regimens consisted of insulin aspart 30/70 in 27 patients (67.5%), lispro 25/75 in 6 (15.0%), lispro 50/50 in 6 (15.0%), and a combination of Insulatard HM and Actrapid HM in 1 patient (2.5%). At baseline, three patients (7.5%) were receiving dulaglutide and one (2.5%) was using basal insulin in addition to premixed insulin; all such agents were discontinued at the time of transition.

**TABLE 1:** Baseline characteristics of the study population.

Variables, mean ± SD or *n* (%)	All patients (*n* = 40)	HbA_1c_ >58 mmol/mol (7.5, *n* = 28)	HbA_1c_ ≤58 mmol/mol (7.5, *n* = 12)	*p*
Age (years)	67.6 ± 10.5	69.0 ± 10.4	64.4 ± 10.4	0.236
Women	19 (47.5)	14 (50.0)	5 (41.7)	0.736
Body height (cm)	160.7 ± 9.0	159.7 ± 8.5	163.2 ± 9.9	0.358
Body weight (kg)	70.7 ± 14.0	70.2 ± 13.3	72.0 ± 16.1	0.827
BMI (kg/m^2^)	27.3 ± 4.6	27.5 ± 4.4	26.9 ± 5.1	0.631
DM duration (years)	18.4 ± 10.0	18.9 ± 9.9	17.4 ± 10.6	0.578
HbA_1c_				
IFCC, mmol/mol	69 ± 22	79 ± 19	46 ± 7	<0.001
NGSP, %	8.5 ± 2.0	9.4 ± 1.7	6.4 ± 0.6	<0.001
FPG				
mmol/L	8.3 ± 2.8	9.0 ± 2.8	6.6 ± 2.0	0.008
mg/dL	148.5 ± 50.7	161.1 ± 51.2	118.9 ± 36.0	0.008
Fasting c‐peptide (ng/mL)	2.1 ± 1.1	2.1 ± 1.2	1.8 ± 1.1	0.766
HOMA‐IR	4.1 ± 1.7	4.3 ± 2.2	3.0 ± 1.0	0.162
Hypoglycaemic incidence	21 (52.5)	9 (32.1)	12 (100.0)	<0.001
Injection treatment				
Premixed insulin only	36 (90.0)	24 (85.7)	12 (100.0)	0.297
Premixed insulin + GLP‐1	3 (7.5)	3 (10.7)	0 (0.0)	0.541
Premixed insulin + Basal	1 (2.5)	1 (3.6)	0 (0.0)	1.000
Premixed insulin dose (units/day)	43.7 ± 22.0	44.9 ± 21.9	40.8 ± 23.0	0.493
Total insulin dose (units/day)	43.9 ± 21.9	45.2 ± 21.7	40.8 ± 23.0	0.439
Injection (times/day)	2.1 ± 0.5	2.1 ± 0.6	2.0 ± 0.0	0.493

*Note*: *p*‐values denotes differences between patients with HbA_1c_ ≥7.5% and <7.5%.

Abbreviations: BMI, body mass index; DM, diabetes mellitus; FPG, fasting plasma glucose; GLP‐1, glucagon‐like peptide‐1; HbA_1c_, glycated haemoglobin; HOMA‐IR, homeostasis model assessment‐insulin resistance index; IFCC, International Federation of Clinical Chemistry and Laboratory Medicine; NGSP, National Glycohaemoglobin Standardisation Programme; SD, standard deviation.

### 3.2 Changes in oral antidiabetic drugs use

OAD use patterns are shown in Table 2. Glinides demonstrated the most substantial increase, with 62.5% newly initiated and 5.0% continued (*p* < 0.001). Biguanide use also increased (15.0% newly initiated, 37.5% continued; *p* = 0.031). Conversely, sodium–glucose cotransporter 2 (SGLT2) inhibitors were markedly reduced (32.5% discontinued; *p* = 0.002), and dipeptidyl peptidase‐4 (DPP‐4) inhibitors were universally discontinued (*p* = 0.002). The use of sulfonylureas, α‐glucosidase inhibitors and thiazolidinediones (TZDs) remained largely unchanged.

**TABLE 2:** Changes in oral antidiabetic drug utilisation before and after transition.

Variables, *n* (%)	Discontinued	Continued	Newly initiated	*p*
Biguanide	0 (0.0)	15 (37.5)	6 (15.0)	0.031
SGLT2 inhibitors	13 (32.5)	3 (7.5)	1 (2.5)	0.002
Sulfonylureas	1 (2.5)	1 (2.5)	1 (2.5)	1.000
Glinide	0 (0.0)	2 (5.0)	25 (62.5)	<0.001
α‐glucosidase inhibitors	2 (5.0)	4 (10.0)	2 (5.0)	1.000
DPP‐4 inhibitors	10 (25.0)	0 (0.0)	0 (0.0)	0.002
TZDs	0 (0.0)	3 (7.5)	0 (0.0)	1.000

Abbreviations: DPP‐4, dipeptidyl peptidase‐4 enzyme; SGLT2, sodium‐glucose cotransporter 2; TZDs, thiazolidinediones.

### 3.3 Efficacy assessment

**FIGURE 1:**
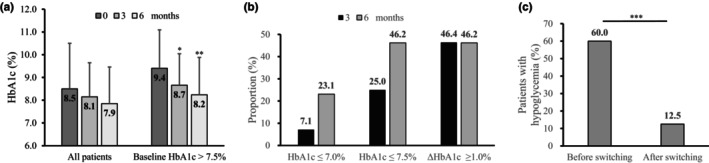
Changes in glycaemic parameters and hypoglycaemia incidence. (a) HbA_1c_ levels at baseline, 3 and 6 months, stratified by baseline HbA_1c_. (b) Proportion of patients achieving HbA_1c_ ≤53 mmol/mol (7.0%), ≤58 mmol/mol (7.5%), or a reduction in HbA_1c_ of at least 11 mmol/mol (1%) at 3 and 6 months in those with baseline HbA_1c_ >58 mmol/mol (7.5%). (c) Percentage of patients experiencing hypoglycaemia episodes before and after transition (all patients). ΔHbA_1c_ was defined as the change in HbA_1c_ from baseline. Data are presented as mean ± standard deviation. *p < 0.05, **p < 0.01, ***p < 0.001 versus baseline. HbA_1c_, glycated haemoglobin.

**FIGURE 2:**
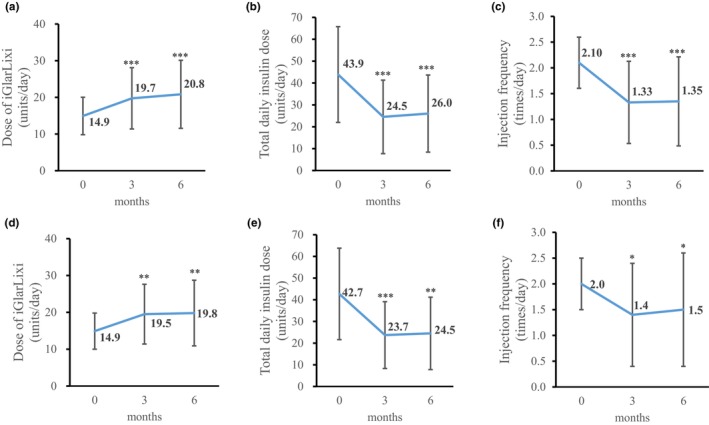
Changes in iGlarLixi dose, insulin dose and injection frequency. Overall population: (a) iGlarLixi dose, (b) total daily insulin dose, and (c) injection frequency. Patients with prior hypoglycaemia (*n* = 24): (d) iGlarLixi dose, (e) total daily insulin dose and (f) injection frequency. Data are presented as mean ± standard deviation. *p < 0.05, **p < 0.01, ***p < 0.001 versus baseline.

Changes in HbA_1c_ and achievement of glycaemic targets are presented in Figure 1. Among patients with baseline HbA_1c_ >58 mmol/mol (7.5%, *n* = 28), HbA_1c_ significantly decreased from 79 ± 18 mmol/mol (9.4 ± 1.7%) at baseline to 71 ± 15 mmol/mol (8.7% ± 1.4%) at 3 months (*p* = 0.03; mean difference [MD] = −8 mmol/mol [95% CI: −16 to −1], −0.7% [95% CI: −1.5% to −0.1%]), and further to 67 ± 18 mmol/mol (8.2% ± 1.6%) at 6 months (*p* = 0.01; MD = −12 mmol/mol [95% CI: −20 to −3], −1.1% [95% CI: −1.8% to −0.3%]). The proportion achieving HbA_1c_ ≤ 58 mmol/mol (7.5%) increased from 25.0% (95% CI: 7.9%–42.1%) at 3 months to 46.2% (95% CI: 25.6%–66.7%) at 6 months, whereas the proportion reaching ≤53 mmol/mol (7.0%) improved from 7.1% (95% CI: −3.0%–17.3%) to 23.1% (95% CI: 5.7%–40.4%) and 46.2% (95% CI: 25.6%–66.7%) achieved a ≥11 mmol/mol (1.0%) reduction in HbA_1c_ from baseline. These glycaemic changes occurred as mean iGlarLixi dose was titrated from 14.9 ± 5.1 units/day (0.22 ± 0.08 unit/kg/day) at initiation to 20.8 ± 9.3 units/day (0.30 ± 0.13 unit/kg/day) at 6 months (*p* < 0.001; MD = 5.9 units/day, 95% CI: 3.6–8.2; Figure 2a; Figure S1a). Meanwhile, the total daily insulin dose declined from 43.9 ± 21.9 units/day (0.63 ± 0.32 unit/kg/day) to 26.0 ± 17.6 units/day (0.37 ± 0.24 unit/kg/day) (*p* < 0.001; MD = −17.9 units/day, 95% CI: −23.5 to −12.2; Figure 2b; Figure S1b) and the average number of daily injections decreased significantly from 2.1 ± 0.5 to 1.4 ± 0.9 times/day at 6 months (*p* < 0.001; MD = −0.8, 95% CI: −1.1 to −0.4; Figure 2c).

### 3.4 Safety assessment

Overall hypoglycaemia incidence decreased substantially, from 60.0% at baseline to 12.5% after transition (*p* < 0.001, Figure 1c). Among patients with hypoglycaemia at baseline, glycaemic control remained stable after switching (FPG: 7.7 ± 2.3 mmol/L [138.7 ± 42.1 mg/dL] at baseline vs. 7.8 ± 3.2 mmol/L [140.7 ± 58.2 mg/dL] at 6 months, *p* = 0.658, MD = 0.1 mmol/L [95% CI: −1.2 to 1.5] or 2.0 mg/dL [95% CI: −22.4 to 26.4]; HbA_1c_: 63 ± 18 mmol/mol [7.9% ± 1.7%] vs. 58 ± 14 mmol/mol [7.4% ± 1.3%], *p* = 0.248, MD = −6 mmol/mol [95% CI: −14 to 3], −0.5% [95% CI: −1.3% to 0.3%]). With glycaemic control maintained, both injection frequency (2.0 ± 0.5 to 1.5 ± 1.1 times/day, *p* = 0.032, MD = −0.6, 95% CI: −1.0 to −0.1; Figure 2f) and total insulin dose (42.7 ± 21.1 units/day [0.62 ± 0.31 unit/kg/day] to 24.5 ± 16.7 units/day [0.36 ± 0.24 unit/kg/day], *p* = 0.001, MD = −18.1, 95% CI: −26.3 to −10.0; Figure 2e; Figure S1d) declined significantly as iGlarLixi was titrated from 14.9 ± 4.9 units/day (0.22 ± 0.09 unit/kg/day) to 19.8 ± 8.9 units/day (0.29 ± 0.13 unit/kg/day) (*p* = 0.004 [0.005], MD = 4.6, 95% CI: 1.8 to 7.5; Figure 2d; Figure S1c).

### 3.5 Subgroup analyses

**FIGURE 3:**
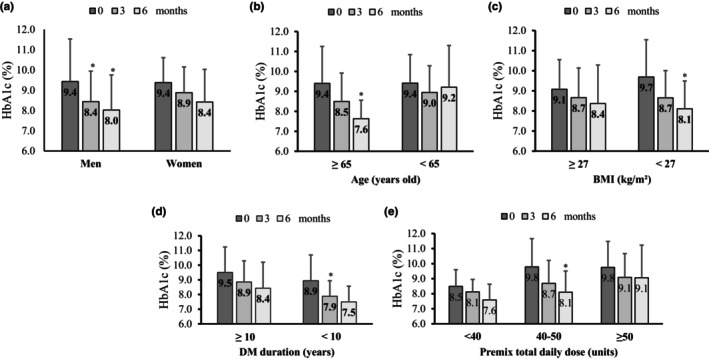
Changes in HbA_1c_ over time in different subgroups with baseline HbA_1c_ >58 mmol/mol (7.5%). Changes in HbA_1c_ stratified by baseline (a) sex, (b) age (≥65 years), (c) BMI (≥27 kg/m^2^), (d) diabetes duration (≥10 years) and (e) premixed insulin total daily dose. Data are presented as mean ± standard deviation. *p < 0.05 versus baseline. BMI, body mass index; DM, diabetes mellitus; HbA_1c_, glycated haemoglobin.

To evaluate whether baseline characteristics influenced treatment response, subgroup analyses were performed in patients with baseline HbA_1c_ >58 mmol/mol (7.5%; Figure 3). In exploratory subgroup analyses, HbA_1c_ reductions were observed within several predefined subgroups, including men, patients aged ≥65 years, those with BMI <27 kg/m^2^, and those with shorter diabetes duration (Men: 79 ± 23 mmol/mol [9.4% ± 2.1%] at baseline to 64 ± 19 mmol/mol [8.0% ± 1.7%] at 6 months, *p* = 0.019, MD = −13 mmol/mol [95% CI: −24 to −2], −1.2% [95% CI: −2.2% to −0.2%]; ≥65 years: 79 ± 21 mmol/mol [9.4% ± 1.9%] to 60 ± 10 mmol/mol [7.6% ± 0.9%], *p* = 0.002, MD = −18 mmol/mol [95% CI: −27 to −8], −1.6% [95% CI: −2.5% to −0.7%]; BMI <27 kg/m^2^: 83 ± 21 mmol/mol [9.7% ± 1.9%] to 65 ± 15 mmol/mol [8.1% ± 1.4%], *p* = 0.039, MD = −16 mmol/mol [95% CI: −29 to −2], −1.4% [95% CI: −2.7% to −0.1%]; diabetes duration <10 years: 74 ± 20 mmol/mol [8.9% ± 1.8%] to 63 ± 12 mmol/mol [7.9% ± 1.1%] at 3 months, *p* = 0.043, MD = −12 mmol/mol [95% CI: −24 to 1], −1.1% [95% CI: −2.2% to 0.1%]). In an exploratory analysis stratified by baseline premixed insulin dose using a 40‐unit cutoff, HbA_1c_ reductions were observed in patients receiving 40–50 units/day (83 ± 21 mmol/mol [9.8% ± 1.9%] to 65 ± 15 mmol/mol [8.1% ± 1.4%], *p* = 0.037, MD = −20 mmol/mol [95% CI: −37 to −3], −1.8% [95% CI: −3.4% to −0.3%]), whereas patients on <40 or ≥50 units/day did not.

### 3.6 Predictors of Glycaemic response

To identify determinants of suboptimal glycaemic response, patients achieving HbA_1c_ ≤58 mmol/mol (7.5%) at 6 months were compared with those who did not (Table 3). HOMA‐IR was significantly higher in the suboptimal response group (4.7 ± 2.5 vs. 3.0 ± 1.0, *p* = 0.030). Variables with *p* < 0.20 in univariate analyses, including baseline HbA_1c_, premixed insulin dose and HOMA‐IR, were entered into a multivariable logistic regression model and adjusted for age and sex. In the multivariable model, HOMA‐IR remained an independent predictor of suboptimal glycaemic response (adjusted odds ratio [OR] 19.94, 95% CI: 1.21–329.90, *p* = 0.037). Men were also associated with a higher likelihood of suboptimal glycaemic response (adjusted OR 197.32, 95% CI: 1.39–27,957.51, *p* = 0.037).

**TABLE 3:** Univariate and multivariate analyses of factors associated with suboptimal glycaemic response (HbA_1c_ >58 mmol/mol [7.5%]) at 6 months.

Variable	Univariate analysis			Multivariate analysis	
	HbA_1c_ levels at 6 months				
	≤58 mmol/mol (7.5%, *n* = 13)	>58 mmol/mol (7.5%, *n* = 12)	*p*	OR (95% CI)	*p*
Sex (men)	6 (46.2)	7 (58.3)	0.695	197.32 (1.39–27957.51)	0.037
Age	66.3 ± 8.8	69.2 ± 10.8	0.611	1.15 (0.95–1.41)	0.161
BMI (kg/m^2^)	26.8 ± 3.9	27.5 ± 3.0	0.936		
DM duration (years)	18.9 ± 8.7	19.6 ± 10.1	0.910		
Baseline HbA_1c_			0.087	1.07 (0.36–3.19)	0.908
IFCC, mol/mol	64 ± 21	78 ± 21			
NGSP, %	8.0 ± 1.9	9.3 ± 1.9			
Premixed insulin (units/day)	37.9 ± 18.5	48.3 ± 20.6	0.186	1.08 (0.99–1.19)	0.096
HOMA‐IR	3.0 ± 1.0	4.7 ± 2.5	0.030	19.94 (1.21–329.90)	0.037

*Note*: Univariate analysis was shown as *n* (%) or mean ± standard deviation.

Abbreviations: BMI, body mass index; CI, confidence interval; DM, diabetes mellitus; HbA_1c_, glycated haemoglobin; HOMA‐IR, homeostasis model assessment‐insulin resistance index; IFCC, International Federation of Clinical Chemistry and Laboratory Medicine; NGSP, National Glycohaemoglobin Standardisation Programme; OR, odds ratio.

## 4 DISCUSSION

This study evaluated the real‐world efficacy and safety of transitioning adults with T2DM from premixed insulin to iGlarLixi. We found that clinically meaningful HbA_1c_ reductions were achieved within 3–6 months among individuals with suboptimal glycaemic control, particularly when iGlarLixi was titrated to fasting glucose targets and accompanied by individualized OAD optimization for postprandial management. In addition, hypoglycaemia incidence, total daily insulin dose, and injection frequency all decreased following the switch, supporting the therapeutic potential, convenience and safety of this de‐escalation strategy. Exploratory subgroup analyses further suggested that older adults, men and individuals with lower BMI may derive greater glycaemic benefit, whereas higher HOMA‐IR independently predicted a suboptimal response, emphasizing the influence of underlying insulin resistance on treatment outcomes.

The glycaemic efficacy of iGlarLixi has been well established in randomized controlled trials evaluating escalation from oral agents, basal insulin, and GLP‐1 RAs, including the LixiLan‐O, LixiLan‐L and LixiLan‐G studies.^10^, ^11^, ^13^ Evidence from Japanese clinical studies, including the LixiLan JP‐O2 trial, has similarly demonstrated effective complementary fasting and postprandial glucose control with iGlarLixi in Asian populations.^14^ However, less is known about transitioning from more complex regimens such as premixed insulin or multiple daily injections (MDIs). Although switching to IDegLira has demonstrated favourable outcomes, substantial heterogeneity in treatment response highlights the need for real‐world evidence across broader populations.^15^

The phase 4 Soli‐SWITCH study provides the most direct comparator for our findings. In that trial, patients transitioning from premixed insulin to iGlarLixi achieved a mean HbA_1c_ reduction of 1.2% (from 8.5% to 7.3%) over 24 weeks, with 37.6% reaching HbA_1c_ <53 mmol/mol (7.0%).^16^ Our findings are consistent with these results and expand them to real‐world settings, which included individuals transitioning not only for poor glycaemic control but also for hypoglycaemia or treatment simplification. This is further supported by the SPARTA Japan real‐world study, a large observational cohort encompassing diverse pre‐switch regimens, which reported significant HbA_1c_ improvement and a low incidence of hypoglycaemia following switching to iGlarLixi in routine clinical care.^17^ Despite having a higher baseline HbA_1c_ in our uncontrolled subgroup compared with the premixed insulin subgroup in Soli‐SWITCH (9.4% vs. 8.5%), the magnitude of HbA_1c_ reduction remained comparable (−13 mmol/mol [−1.2%]). The lower proportion achieving HbA_1c_ ≤53 mmol/mol (7.0%; 23.1% vs. 37.6% in Soli‐SWITCH) likely reflects greater clinical heterogeneity and comorbidity burden in real‐world practice relative to controlled trial environments.^16^

A notable difference between our study and Soli‐SWITCH was the change in total daily insulin dose. Whereas Soli‐SWITCH reported minimal net change (+2.5 units/day), our cohort experienced a substantial reduction (−17.9 units/day; *p* < 0.001).^16^ Several factors may explain this discrepancy. First, the Soli‐SWITCH cohort had a higher mean BMI (29.4 vs. 27.3 kg/m^2^), suggesting greater insulin resistance and therefore a higher insulin requirement. Second, short‐acting insulin secretagogues, particularly glinides, were commonly initiated in our cohort and may have enhanced postprandial glucose control without increasing total insulin requirements, a strategy not specified in the Soli‐SWITCH protocol.^16^, ^18^ Moreover, Soli‐SWITCH did not describe longitudinal OAD modifications, whereas we systematically categorized OAD adjustments (discontinued, continued, or newly initiated), revealing reduced reliance on SGLT2 inhibitors and increased use of glinides. These findings suggest that clinicians may preferentially augment postprandial‐directed therapies when intensifying treatment with iGlarLixi.

The marked decline in hypoglycaemia incidence (60.0% at baseline to 12.5% post‐transition) highlights the safety advantages of switching from premixed insulin to iGlarLixi. Importantly, during the 6‐month follow‐up, no patients discontinued iGlarLixi due to hypoglycaemia, and reported hypoglycaemic events were successfully managed with patient education and temporary dose reduction, with subsequent improvement. Soli‐SWITCH reported an overall hypoglycaemia rate of 38.3%, but did not distinguish pre‐ versus post‐switch events, limiting comparability.^16^ Premixed insulin contains a rapidly peaking prandial component and an intermediate‐acting fraction with substantial overlap (“shoulder effect”), predisposing patients to delayed postprandial hypoglycaemia.^19^ In contrast, iGlarLixi provides a stable basal insulin profile together with glucose‐dependent postprandial control via lixisenatide, which slows gastric emptying and reduces glucagon secretion without stimulating insulin release during low‐glucose states.^20^ This pharmacologic profile likely contributed to the improved safety observed in our study, particularly among patients with baseline hypoglycaemia who experienced significant reductions in injection burden and total insulin dose without deterioration in glycaemic control. These findings align with the SoliMix trial, in which iGlarLixi achieved higher rates of hypoglycaemia‐free glycaemic target attainment and improved treatment satisfaction compared with BIAsp 30.^21^, ^22^ Although we did not assess treatment satisfaction or weight changes, prior studies have shown that reductions in injection burden and insulin dose are associated with improved adherence and favourable weight trajectories.^23^, ^24^, ^25^, ^26^ Taken together, these results suggest that iGlarLixi may be a safe and well‐tolerated alternative to premixed insulin, especially for individuals at elevated hypoglycaemia risk.

Exploratory subgroup analyses further identified several baseline factors associated with differential glycaemic response. Greater HbA_1c_ reductions were seen in men, older adults (≥65 years), individuals with BMI <27 kg/m^2^, and those with diabetes duration <10 years. Older adults may demonstrate better adherence, while individuals with higher BMI may require higher insulin doses or additional therapies due to more severe insulin resistance.^18^, ^27^ Consistent with this notion, HOMA‐IR emerged as an independent predictor of suboptimal response. Because all patients were receiving exogenous insulin, we employed a C‐peptide‐based modified HOMA‐IR to estimate endogenous insulin resistance.^12^ Although this method avoids confounding by injected insulin, it has not been extensively validated in insulin‐treated populations; therefore, our findings should be viewed as exploratory.^28^

Interestingly, patients previously receiving 40–50 units/day of premixed insulin demonstrated the largest HbA_1c_ improvement following transition to iGlarLixi. This likely reflects an optimal alignment between prior insulin requirements and the dose ceiling of iGlarLixi in Taiwan, where only the 10–40 SoloStar pen is available.^29^, ^30^ Patients requiring >50 units/day may experience under‐dosing, while those using <40 units/day receive submaximal lixisenatide exposure at the lower dose steps. These observations generate hypotheses regarding patient characteristics associated with glycaemic response but require confirmation in larger studies with formal interaction testing.

This real‐world study included patients with diverse clinical indications for transitioning to iGlarLixi and incorporated detailed descriptions of OAD adjustments and subgroup outcomes, offering insights beyond those reported in clinical trials. Nonetheless, its retrospective design, small sample size, absence of a comparator group, incomplete assessment of weight change and treatment satisfaction, and reliance on routine clinical data without CGM or complete SMBG profiles limited causal inference and the generalizability of subgroup findings.

In conclusion, iGlarLixi appears to be a feasible and safe de‐escalation option for adults with T2DM treated with premixed insulin in real‐world practice. Transitioning to iGlarLixi was associated with simplified injection schedules, reduced insulin burden, improved glycaemic outcomes and a lower risk of hypoglycaemia. Insulin resistance may be an important determinant of treatment response in such transitions.

## FUNDING INFORMATION

This research was partially supported by funding from Taipei Veterans General Hospital (V112B‐003, V113C‐027 and V115D85‐001‐MY2‐1) and the National Science and Technology Council, Taiwan (112‐2314‐B‐A49‐081 and 114‐2314‐B‐075‐044‐MY3).

## CONFLICT OF INTEREST STATEMENT

The authors declare no conflict of interest.

## Supporting information


**Figure S1.** Changes in weight‐adjusted iGlarLixi and insulin dose. Overall population: (a) iGlarLixi dose and (b) total daily insulin dose. Patients with prior hypoglycaemia (*n* = 24): (c) iGlarLixi dose and (d) total daily insulin dose. Data are presented as mean ± standard deviation. **p < 0.01, ***p < 0.001 versus baseline.

## Data Availability

The data that support the findings of this study are available from the corresponding author upon reasonable request.
