# Supplementary figures and images for: Efficacy and safety of switching to iGlarLixi from premixed insulin therapy in patients with type 2 diabetes: A real‐world experience
Source: Diabet Med. 2026 Apr 1;43(7):e70275. doi: 10.1111/dme.70275 (PMC13257900; doi:10.1111/dme.70275)

## Slide 1
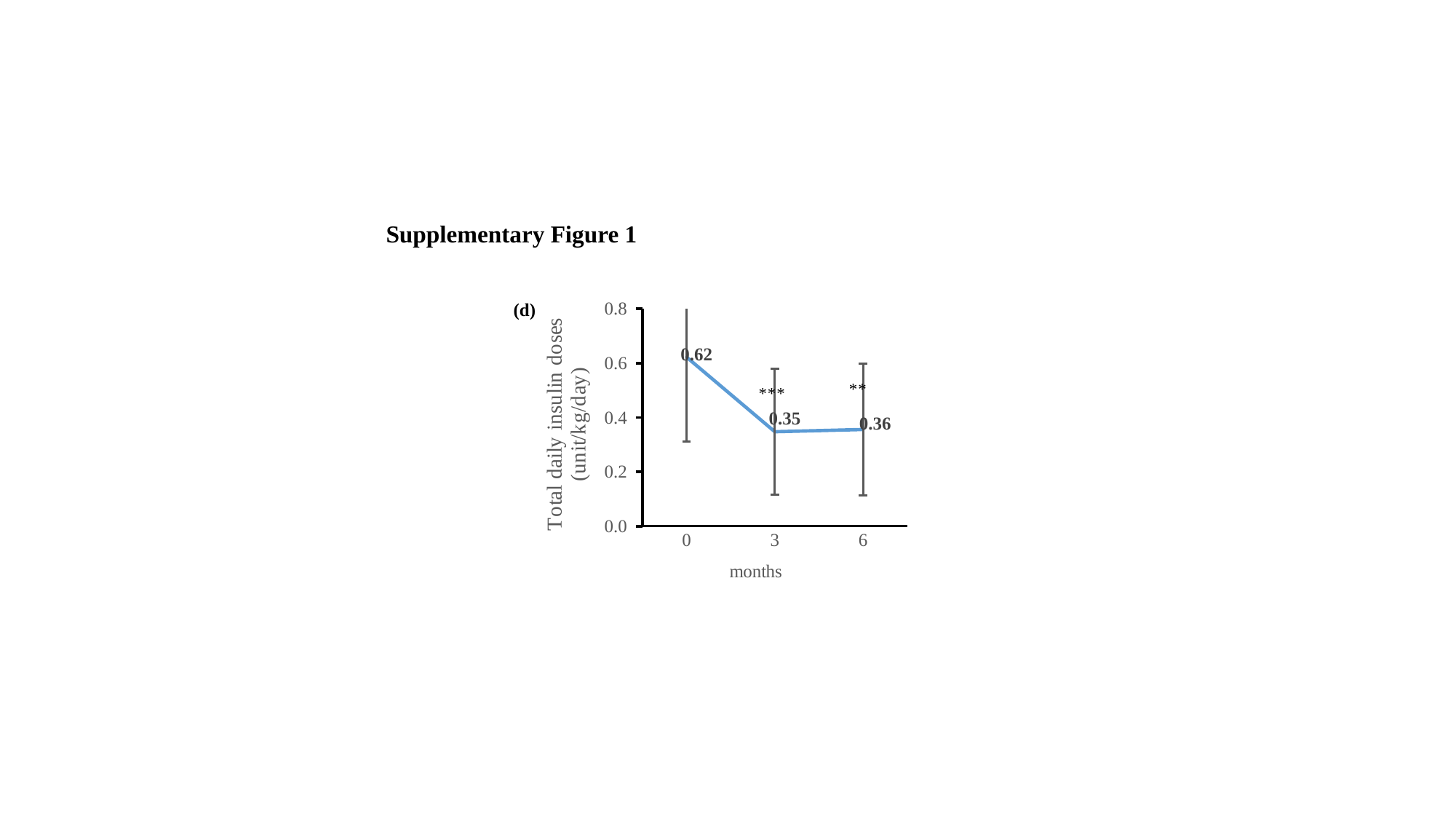

Supplementary Figure 1
(d)
### Chart
| Category | isulin units/kg |
|---|---|
| 0 | 0.6221 |
| 3 | 0.3478 |
| 6 | 0.356 |

Supplement: Supplementary file 1 — Figure S1. Changes in weight‐adjusted iGlarLixi and insulin dose. Overall population: (a) iGlarLixi dose and (b) total daily insulin dose. Patients with prior hypoglycaemia (n = 24): (c) iGlarLixi dose and (d) total daily insulin dose. Data are presented as mean ± standard deviation. **p < 0.01, ***p < 0.001 versus baseline. [file DME-43-e70275-s001.zip › dme70275-sup-0004-FigureS1-S1@Figure S1(d).pptx]

## Slide 1
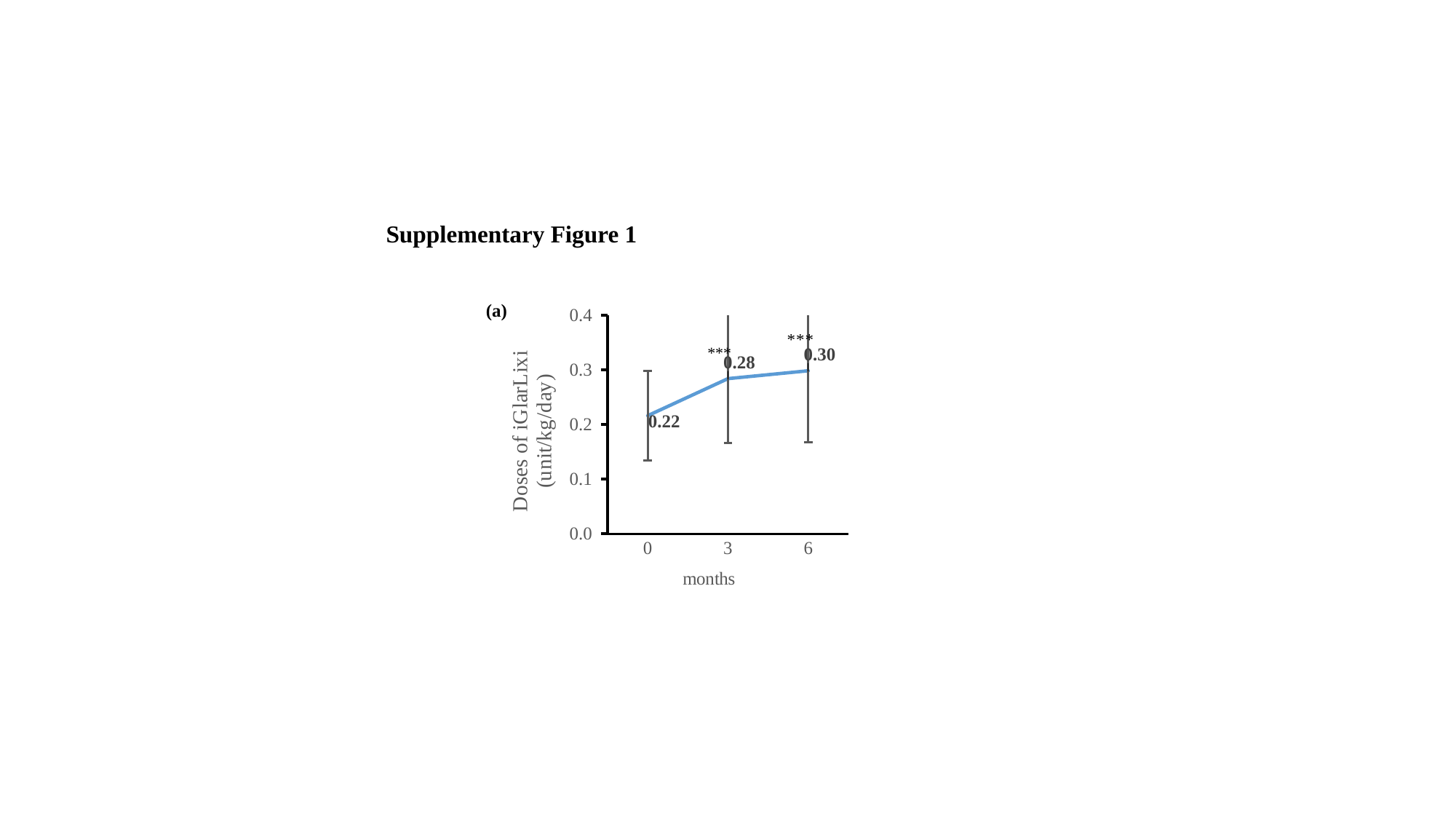

Supplementary Figure 1
(a)
### Chart
| Category | mean |
|---|---|
| 0 | 0.2161 |
| 3 | 0.2839 |
| 6 | 0.2983 |

Supplement: Supplementary file 1 — Figure S1. Changes in weight‐adjusted iGlarLixi and insulin dose. Overall population: (a) iGlarLixi dose and (b) total daily insulin dose. Patients with prior hypoglycaemia (n = 24): (c) iGlarLixi dose and (d) total daily insulin dose. Data are presented as mean ± standard deviation. **p < 0.01, ***p < 0.001 versus baseline. [file DME-43-e70275-s001.zip › dme70275-sup-0001-FigureS1-S1@Figure S1(a).pptx]

## Slide 1
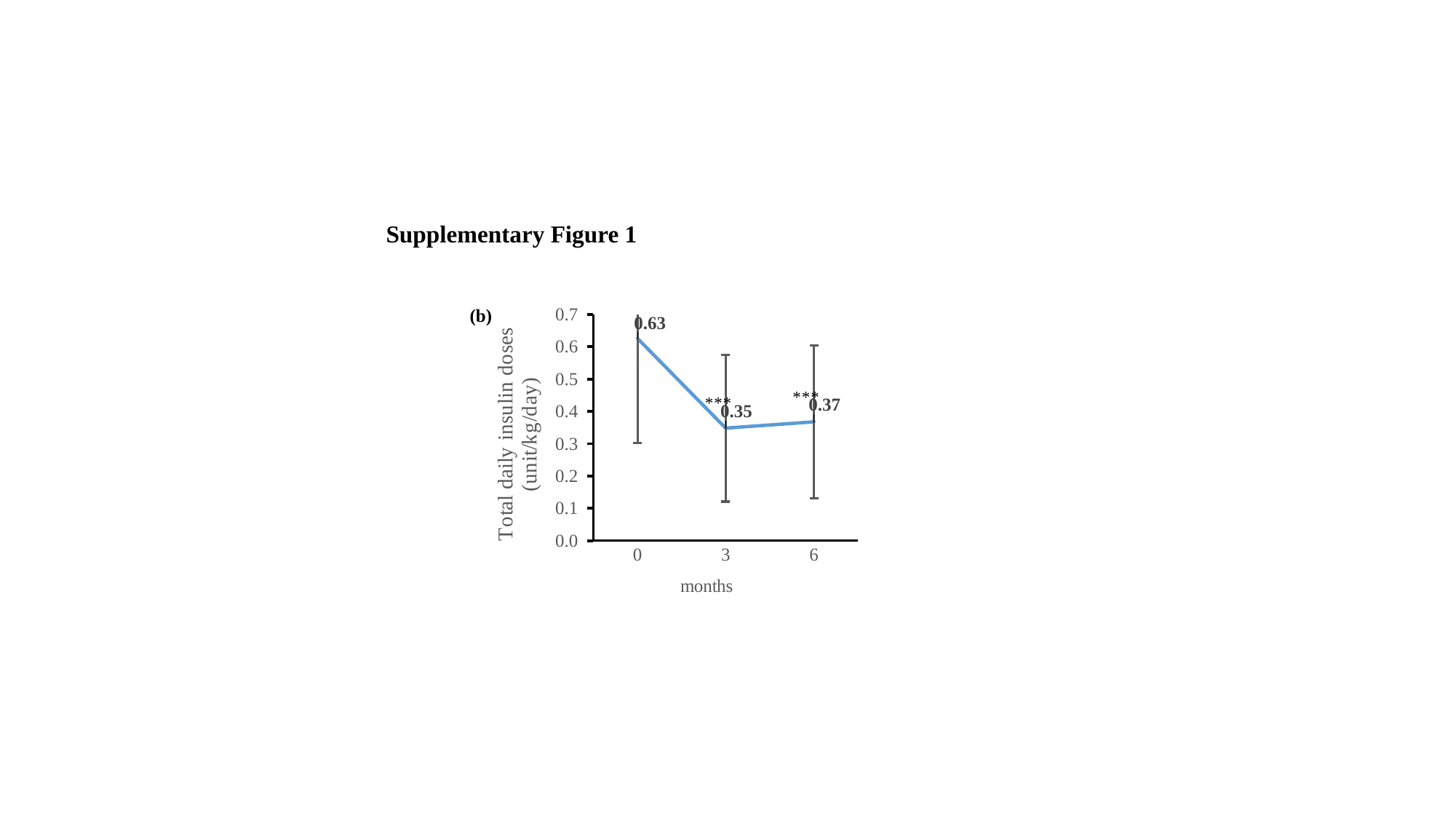

Supplementary Figure 1
### Chart
| Category | mean |
|---|---|
| 0 | 0.6257 |
| 3 | 0.3483 |
| 6 | 0.3678 |(b)

Supplement: Supplementary file 1 — Figure S1. Changes in weight‐adjusted iGlarLixi and insulin dose. Overall population: (a) iGlarLixi dose and (b) total daily insulin dose. Patients with prior hypoglycaemia (n = 24): (c) iGlarLixi dose and (d) total daily insulin dose. Data are presented as mean ± standard deviation. **p < 0.01, ***p < 0.001 versus baseline. [file DME-43-e70275-s001.zip › dme70275-sup-0002-FigureS1-S1@Figure S1(b).pptx]

## Slide 1
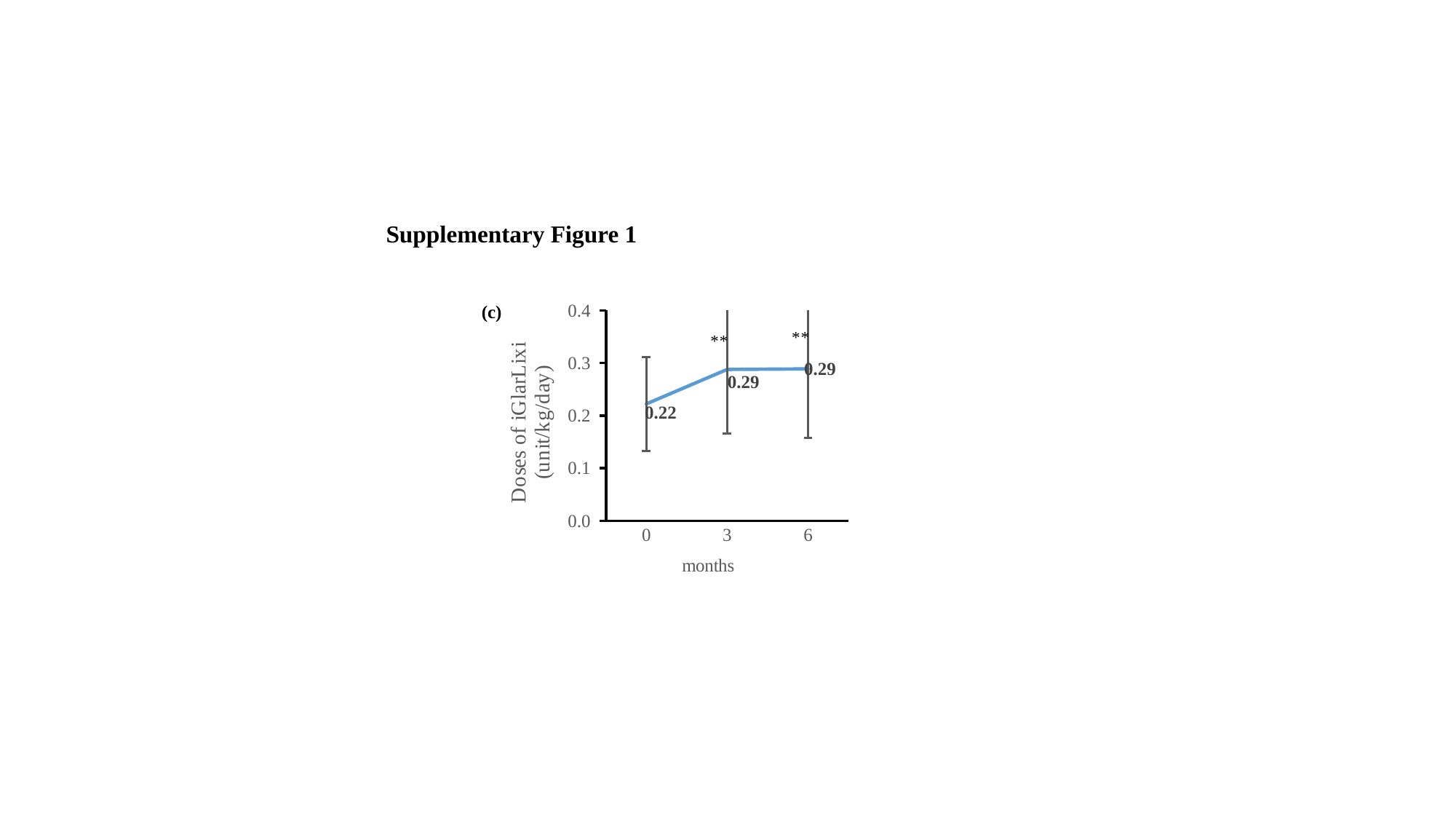

Supplementary Figure 1
### Chart
| Category | iGlarLixi dose/kg |
|---|---|
| 0 | 0.2223 |
| 3 | 0.2878 |
| 6 | 0.2889 |(c)

Supplement: Supplementary file 1 — Figure S1. Changes in weight‐adjusted iGlarLixi and insulin dose. Overall population: (a) iGlarLixi dose and (b) total daily insulin dose. Patients with prior hypoglycaemia (n = 24): (c) iGlarLixi dose and (d) total daily insulin dose. Data are presented as mean ± standard deviation. **p < 0.01, ***p < 0.001 versus baseline. [file DME-43-e70275-s001.zip › dme70275-sup-0003-FigureS1-S1@Figure S1(c).pptx]
